# Diagnosing, imaging, and successfully treating a debilitating case of Bing–Neel syndrome: A multidisciplinary feat

**DOI:** 10.1002/ccr3.7296

**Published:** 2023-05-04

**Authors:** Robert N. Kerley, Niamh O'Donnell, Fiona Lynott, Riona Mulcahy, Brian Hennessy

**Affiliations:** ^1^ Cork University Hospital Cork Ireland; ^2^ University Hospital Waterford Waterford Ireland

**Keywords:** Bing–Neel syndrome, diagnostic imaging, Waldenstrom macroglobulinemia

## Abstract

**Key Clinical Message:**

We present a case of Bing–Neel syndrome a rare central nervous system lymphoplasmocytic lymphoma associated with Waldenstrom macroglobulinemia. Diagnosis should be considered in the context of unexplained neurological symptoms in the presence of macroglobulinemia.

**Abstract:**

Waldenstroms macroglobulinaemia (WM) is a rare B‐cell lymphoma representing ~2% of all hematological malignancies. While most neurological complications of WM are secondary to the overproduction of immunoglobulin M (IgM), Bing‐Neel syndrome (BNS) is an extremely rare direct central nervous system (CNS) infiltration by malignant lymphoplasmocytic lymphoma (LPL) cells. Limited information on BNS exists in the literature with sparse case reports and case series. Here, we present a diagnostically challenging BNS case successfully treated with systemic chemoimmunotherapy and ibrutinib, with remarkable clinical response.

## 1 INITIAL PRESENTATION

A 63‐year‐old lady presented to the emergency department with a 1‐day history of binocular diplopia and evidence of abducens nerve palsy on examination. Medical history included dyslipidemia, non‐alcoholic steatohepatosis, and colonic poly removal. Visual symptoms occurred gradually over weeks without diurnal variation. Fundal examination was unremarkable. Admission bloods, chest x‐ray, and CT brain did not reveal a cause for the patient's neurological symptoms. Full blood count showed a hemoglobin of 10.6 g/dL but was otherwise unremarkable. Her vasculitic, viral, and lyme serology screen were negative. Incidentally, her serum protein electrophoresis showed an immunoglobulin M (IgM) paraproteinemia, with a paraprotein level of 4.2 g/L. Renal function, serum calcium, beta‐2‐micorglobulin, and plasma viscosity levels were within normal ranges. An MRI Brain, orbits and cervical cord revealed significant cervical spondylosis with multilevel nerve root impingement. Lumbar puncture (LP) showed raised cerebrospinal fluid (CSF) protein (0.97 g/dL) and leukocyte count (31 × 10^9^/L, 90% lymphocytes, 10% polymorphs). She was discharged with an incidental diagnosis of monoclonal gammopathy of undetermined significance with close follow from neurology and general medicine.

## 2 PROGRESSION AND DIAGNOSIS

**FIGURE 1:**
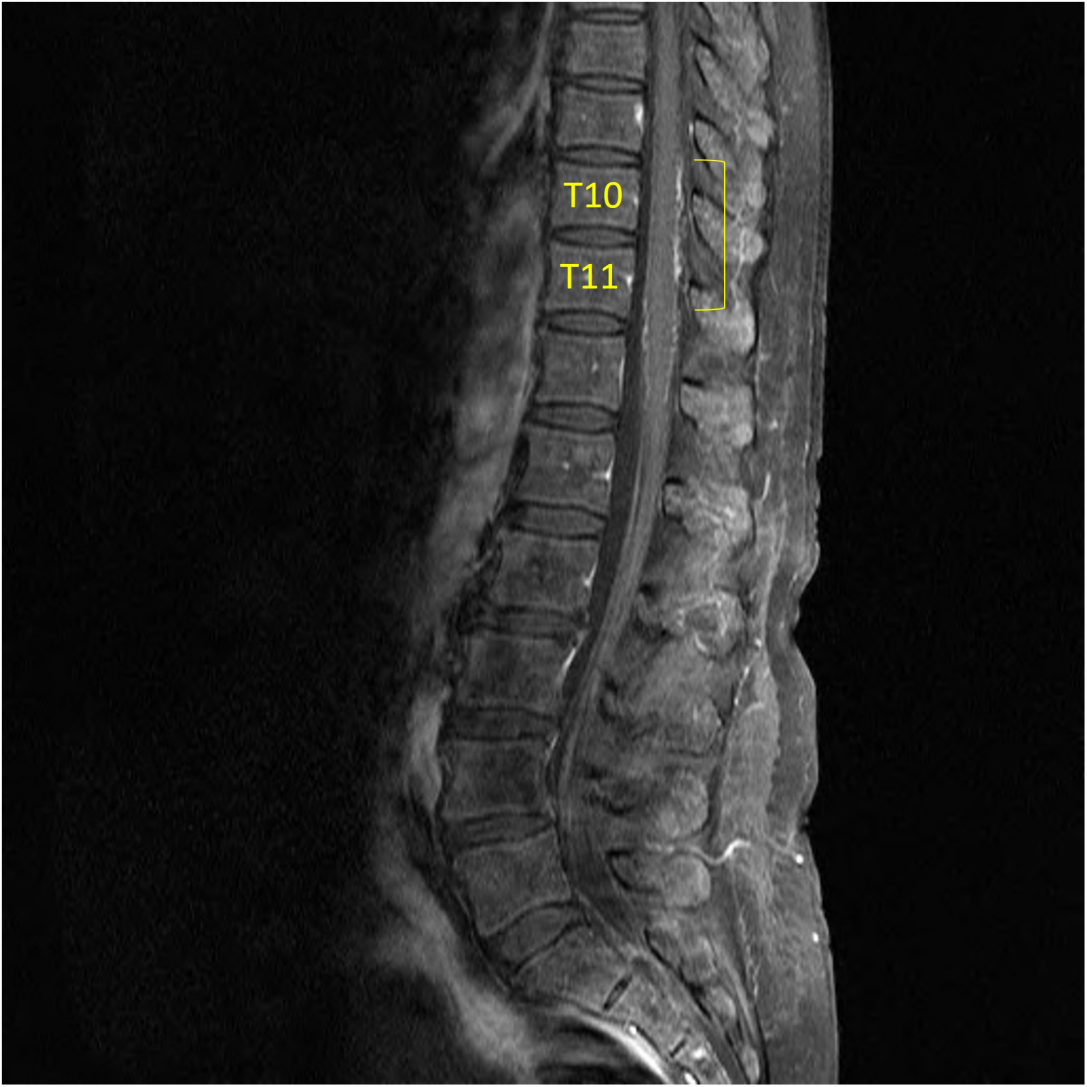
MRI whole spine outlining subtle posterior meningeal enhancement at T10 and T11.

Two weeks later the patient was readmitted with ataxia, gait disturbance, and loss of power bilaterally in her lower limbs. On examination, power was 3/5 in both lower limbs and 5/5 in her upper limbs. Tone was normal, sensation, and reflexes were intact without evidence of coordination deficits and an extensor plantar reflex. A repeat MRI brain and whole spine with contrast was negative for leptomeningeal or parenchymal enhancement. Without a diagnosis and progressive neurological deficits, a broader differential was conducted to include nerve conduction studies, a repeat autoimmune, vasculitic and viral screen, CT thorax, abdomen and pelvis, and serum ACE all of which were non‐diagnostic. Finally, a repeat LP with CSF immunophenotyping and second MRI whole spine with contrast were ordered to exclude infiltrative disease (Figure 1). This MRI result was discussed at three major neuroradiology sites in our country to form a consensus in the presence of infiltrative disease. Subtle posterior meningeal enhancement involving the distal cord at T10 and T11 vertebral bodies was observed. In the context of macroglobulinemia, a bone marrow biopsy was performed which showed a normocellular specimen with no evidence of lymphomatous infiltration. While awaiting her CSF sample to return from flow cytometry she deteriorated with ascending paralysis compromising respiratory drive and requiring transfer to the intensive care unit. The following morning, CSF immunphenotyping revealed a clonal population of kappa light chains restricted B cells, which were CD5 and CD10 negative, consistent with CNS involvement by lymphoma, in keeping with a diagnosis of Bing–Neel syndrome (BNS).

## 3 TREATMENT

Prior to diagnosis, intravenous (IV) methylprednisolone 1 g was given for 3 days with a partial subjective response to limb paralysis but no response as regards her abducens nerve palsy. Following CSF analysis, a combination of chemo‐ and immunotherapy with intrathecal (IT) methotrexate (MTX), subcutaneous rituximab, and pulsed oral dexamethasone were urgently commenced. Twenty‐four hours post‐IT‐MTX, ibrutinib 420 mg PO OD was introduced. Due to the patient's neurological deficits and difficulty performing LPs, as well as reluctance to interrupt ibrutinib for LPs, a decision was made to switch from IT‐MTX to high‐dose IV MTX (3.5 g/m^2^) for second and third cycles. In total the patient received the following induction therapy: one IT MTX, two cycles of high‐dose IV MTX, weekly rituximab for 4 weeks, four pulses of oral dexamethasone (40 mg fortnightly), and oral ibrutinib. This treatment was well tolerated. A repeat CSF sample was sent for flow cytometry post the above treatment with no clonal population of B cells detected. In addition, the patient's neurological symptoms responded over weeks from 1/5 power to 4/5 power in her lower limbs. As per the 2014 task force guidelines on BNS, these CSF findings in conjunction with the resolution of symptoms suggests a clinical response in keeping with complete remission.^1^ The patient received two further doses of subcutaneous rituximab monthly. She remained on oral ibrutinib for 18 months and unfortunately relapsed. She is currently undergoing treatment with daratumumab‐based chemotherapy.

## 4 DISCUSSION

The clinical presentation of BNS as shown in this case is diverse and non‐specific. A critical point that could explain the under recognition of BNS is its frequent occurrence independent of any systemic progression of Waldenstrom macroglobulinemia (WM0), with up to one‐third of BNS cases coinciding with diagnosis of WM.^2^, ^3^ Radiological assessment was critical to the diagnosis in this case requiring input from experienced neuroradiologists. In a case series of 24 patients, the most common MRI finding was leptomeningeal infiltration either intracranial or spinal with a prevalence reaching 70.8%.^4^ Dural and parenchymal involvement were present in 37.5% and 41.7% of patients, respectively. Autopsy results have observed more extensive meningeal and perivascular infiltration by malignant cells than that revealed by MRI.^5^ the gold standard for diagnosis is a histological biopsy of the affected area or CSF analysis by flow cytometry demonstrating malignant cells as previously outlined.^1^

There is no established treatment regimen for BNS to date. Patients typically receive a combination of systemic and IT chemotherapy, immunotherapy, and novel agents, such as ibrutintib, an oral Bruton kinase inhibitor. The goal of treatment should be to (1) reverse the patient's clinical symptoms and (2) induce prolonged progression‐free survival.^6^ Remission has been reported with both IT therapies and/or systemic chemotherapies.^1^, ^2^, ^7^, ^8^ Use of high‐dose MTX is extrapolated from data in primary CNS lymphoma while direct evidence supports the use of rituximab in BNS.^3^, ^9^ Ibrutinib has been increasingly employed in BNS treatment as well as in other primary CNS lymphomas.^10^, ^11^, ^12^ Data suggest ibrutinib maintenance also has a role in prolonging progression‐free survival rationalizing its use in our patient's case.^13^ In one review of 34 patients with BNS, the estimated overall survival rate at 3 years was 59%.^14^ Age > 65 years, thrombocytopenia and previous treatment for WM were all associated with worse prognosis.^14^

## 5 CONCLUSION

In conclusion, BNS is a rare infiltrative CNS lymphoplasmocytic lymphoma associated with WM. It's presentation is highly variable and this case illustrates the need for multidisciplinary input, appropriate imaging, and CSF analysis during the work up of unexplained neurological symptoms in the presence of macroglobulinemia.

## AUTHOR CONTRIBUTIONS

**Robert Kerley:** Conceptualization; data curation; formal analysis; methodology; writing – original draft; writing – review and editing. **Niamh O’Donnell:** Data curation. **Fiona Lynott:** Conceptualization; data curation; formal analysis; methodology; writing – original draft. **Riona Mulcahy:** Supervision. **Brian Hennessy:** Supervision.

## FUNDING INFORMATION

This case report did not receive funding.

## CONFLICT OF INTEREST STATEMENT

The authors have no conflict of interest to declare.

## CONSENT

Written informed consent was obtained from the patient to publish this report in accordance with the journal′s patient consent policy.

## Data Availability

Data openly available in a public repository that issues datasets with DOIs.
